# Canonical Correlation Analysis for Gene-Based Pleiotropy Discovery

**DOI:** 10.1371/journal.pcbi.1003876

**Published:** 2014-10-16

**Authors:** Jose A. Seoane, Colin Campbell, Ian N. M. Day, Juan P. Casas, Tom R. Gaunt

**Affiliations:** 1 School of Social and Community Medicine, University of Bristol, Bristol, United Kingdom; 2 Intelligent Systems Laboratory, University of Bristol, Bristol, United Kingdom; 3 Department of Non-communicable Disease Epidemiology, London School of Hygiene and Tropical Medicine, London, United Kingdom; 4 MRC Integrative Epidemiology Unit, University of Bristol, Bristol, United Kingdom; The Centre for Research and Technology Hellas, Greece

## Abstract

Genome-wide association studies have identified a wealth of genetic variants involved in complex traits and multifactorial diseases. There is now considerable interest in testing variants for association with multiple phenotypes (pleiotropy) and for testing multiple variants for association with a single phenotype (gene-based association tests). Such approaches can increase statistical power by combining evidence for association over multiple phenotypes or genetic variants respectively. Canonical Correlation Analysis (CCA) measures the correlation between two sets of multidimensional variables, and thus offers the potential to combine these two approaches. To apply CCA, we must restrict the number of attributes relative to the number of samples. Hence we consider modules of genetic variation that can comprise a gene, a pathway or another biologically relevant grouping, and/or a set of phenotypes. In order to do this, we use an attribute selection strategy based on a binary genetic algorithm. Applied to a UK-based prospective cohort study of 4286 women (the British Women's Heart and Health Study), we find improved statistical power in the detection of previously reported genetic associations, and identify a number of novel pleiotropic associations between genetic variants and phenotypes. New discoveries include gene-based association of *NSF* with triglyceride levels and several genes (*ACSM3*, *ERI2*, *IL18RAP*, *IL23RAP* and *NRG1*) with left ventricular hypertrophy phenotypes. In multiple-phenotype analyses we find association of *NRG1* with left ventricular hypertrophy phenotypes, fibrinogen and urea and pleiotropic relationships of *F7* and *F10* with Factor VII, Factor IX and cholesterol levels.

## Author Summary

Pleiotropy appears when a variation in one gene affects to several non-related phenotypes. The study of this phenomenon can be useful in gene function discovery, but also in the study of the evolution of a gene. In this paper, we present a methodology, based on Canonical Correlation Analysis, which studies gene-centered multiple association of the variation of SNPs in one or a set of genes with one or a set of phenotypes. The resulting methodology can be applied in gene-centered association analysis, multiple association analysis or pleiotropic pattern discovery. We apply this methodology with a genotype dataset and a set of cardiovascular related phenotypes, and discover new gene association between gene NRG1 and phenotypes related with left ventricular hypertrophy, and pleiotropic effects of this gene with other phenotypes as coagulation factors and urea or pleiotropic effects between coagulation related genes F7 and F10 with coagulation factors and cholesterol levels. This methodology could be also used to find multiple associations in other omics datasets.

## Introduction

Pleiotropy refers to a phenomenon in which a single locus affects two or more apparently unrelated phenotypic traits. It is often identified as a single mutation that affects these two or more wild-type traits [1]. The study of pleiotropic genes usually involves the mapping of phenotypic traits to a single mutant locus. When two or more traits consistently segregate with a particular mutation, this mutation is then classified as pleiotropic. In the case of *S. cerevisiae* (yeast), it has been argued that the pleiotropic effects of a gene are not usually conferred by multiple molecular functions of the gene, but by multiple consequences (biological processes) of a single molecular function [2]. Tyler *et al.* [3] defined the concept of vertical and horizontal pleiotropy, extending the definition of relational and mosaic pleiotropy proposed by Hadorn and Mittwoch [4]. Vertical or relational pleiotropy appears when a mutation in one gene produces a modification of one particular phenotype, which leads to modification in one or several related phenotypes. By contrast, horizontal or mosaic pleiotropy appears when one mutation in one gene with a causal implication in several biological mechanisms, causes a disruption in these mechanisms. This causes alteration in very different phenotypes, which are observable at the same physiological level. Some papers [5] [6] [7] have established a high level of pleiotropy for certain genes, particularly genes associated with disease [8].

To discover such associations we could use a range of multivariate techniques which highlight the dependence of a single variable on a set of independent variables. Some proposals are based on combining univariate association measures for different phenotypes in order to find pleiotropic effects, such as *PRIMe* [9] or Yang *et al*'s approach [10], based in O'Brien's method [11]. An approach taken by Li [5], uses Fisher's combined p-value approach [12], adjusting Fisher's combined measure using a Satterwhite approximation method. Other approaches use a Bayesian network approach [13] or multiple regression analysis [14]. However, for the purposes of pleiotropy analysis, we are most interested in finding dependencies between two multivariate sets of variables, rather than a relation of one set with one dependent variable. Various techniques have also been introduced to deal with such multivariate problems [15]. An example of this multiple SNP/multiple phenotype analysis is *GUESS* [16], which is an implementation of a Bayesian variable selection algorithm for multiple regression using evolutionary Monte Carlo techniques: the algorithm selects relevant SNPs and identifies the contribution of each SNP to single or multiple traits.

In this paper, we will focus on Canonical Correlation Analysis (CCA) [17], which uses linear combinations of variables derived from two sets of data objects and finds those combinations which are maximally correlated with each other. The variables found in the first iteration of the method give the first set of *canonical variables*. In subsequent iterations we seek variables which maximize the same correlation function, subject to the constraint that they are uncorrelated with previous sets of canonical variables.

CCA has been used as an efficient and powerful approach for both univariate and multivariate gene-based association tests. For genomic multivariate data analysis, such an approach would involve finding linear combinations over very large blocks of features, typically involving tens of thousands of features. However, to use CCA, the number of samples should be more than the number of features. To handle this issue, some solutions have been proposed for genomic data integration, such as sparse CCA [18]. With this approach, sparsity is intrinsically achieved by the algorithm so that the number of features used is less than the sample size. This method maximizes the correlations between these selected subsets using a regularization procedure similar to LASSO. Adaptive SCCA [19] selects fewer features which are more correlated and Waaijenborg *et al.* [20], propose a method called penalized CCA to find associations between gene expression and copy number variation data. Other variants on CCA which are applicable include non-linear extensions of CCA, such as kernel CCA [21], [22], Bayesian approaches to CCA [23], [24] and sparse CCA models for handling more than two types of data [25].

CCA for association analysis was proposed initially by Ferreira and Purcel [26] and subsequently extended [27]. Both these papers apply CCA to multiple trait/single genotype analysis (pleiotropy analysis), while the latter also considers the case of several markers (gene centered pleiotropy analysis) and several traits, or several markers and one trait (epistasis analysis). Since the original publication [26], CCA has been used for multiple association analysis elsewhere, including a single SNP, multiple phenotype association approach [28] to analyze blood phenotypes related with metabolic syndrome in mice, and use of a sparse version of CCA to discover associations between single locus and multiple neuroimage phenotypes [29]. Further applications of CCA include a study [26] of pleiotropy in white cell related traits using a single locus/multiple trait approach, and use of CCA for single SNP/multiple trait analysis to find different child behavior profiles [30].

In this paper we propose an alternative approach for using CCA in which we select feature sets via biological insight, based on association with a gene, a pathway or another biologically relevant grouping. As detailed below, to maximize the association between genetic data and different phenotypes, we combine the CCA approach proposed by Ferreira and Purcell [26] with an optimization technique, drawn from integer programming. We will refer to any discovered significant associations between subsets of the genetic and phenotype data as putative *association rules*.

## Results

Our results are divided into (a) single gene/single phenotype; (b) single gene/multiple phenotype (in which the algorithm identifies the set(s) of phenotypes associated with a single gene); (c) multiple gene/single phenotype (in which the algorithm identifies the set(s) of genes associated with a single phenotype); (d) multiple gene/multiple phenotype (in which the algorithm selects sets of both genes and phenotypes that correlate).

### Single gene/single phenotype analysis

**Figure 1:**
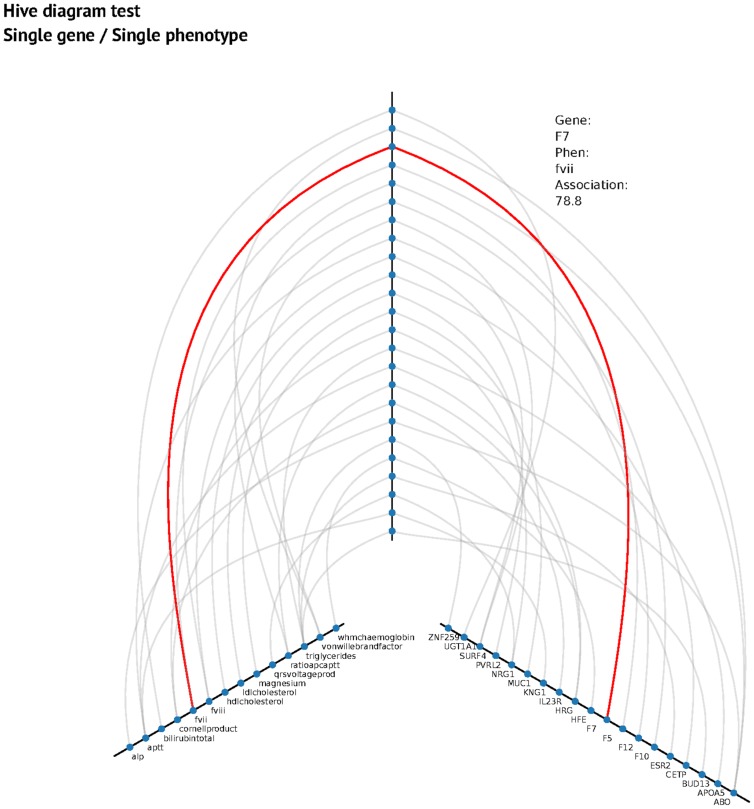
Hive plot for single gene/single phenotype. The vertical axis represents the association value (higher, more association). The left axis represents phenotypes and the right axis represents genes. An interactive hive plot is published on the project webpage (http://pleioexp.epi.bris.ac.uk/cca/1gene1phenhive.html).

This approach consists of a gene centered association analysis with each single phenotype using simple CCA without any search heuristic. It is exactly the same approach used previously by Tang and Ferreira [27], consisting of a multiple association of all the SNPs close to a gene (see Methods for more detail) with a particular phenotype. In order to correct for multiple testing, we use a Bonferroni correction for 3648 genes and 82 phenotypes, giving a “threshold” p-value of 1.67×10^−06^ corresponding to p = 0.05 for a single test. We found 62 genes with significant association (p<1.67×10^−06^). Most of the time this association reflects the most associated SNP in a gene. The most important associations are presented in the hive plot in Figure 1.

In Table 1 we show some of the associations found and publications that supports these findings. All such associations can be found in Table S1, where we compare the association values between this approach and conventional single SNP association tests.

**Table 1:** Single gene/single phenotype association.

Gene	Entrez gene#	Phenotype	P-value	Publication
*TRIM46*	80128	Serum Magnesium	4.63E-010	Not Reported
*MUC1*	4582	Serum Magnesium	1.37E-014	20700443
*UGT1A family*	7361, 54577, 54575, 54600, 54579, 54657	Bilirubin	1.29E-112	19419973,21646302
*ABO*	28	Von Willebrand Factor	9.43E-112	21534939
*ABO*	28	Factor VIII	2.29E-077	23381943
*ABO*	28	ALP	4.35E-012	18940312
*F7*	2155	Factor VII	1.49E-079	17903294
*F5*	2153	Ratio APC/APTT	2.77E-062	23188048
*F10*	2159	Factor VII	1.24E-060	17903294
*SURF4*	6836	Von Willebrand Factor	5.66E-037	21534939
*SURF4*	6836	Factor VIII	1.57E-024	Not Reported
*SURF4*	6836	ALP	3.10E-009	Not Reported
*F12*	2161	APTT	5.77E-035	23188048
*PVRL2*	5819	LDL cholesterol	2.26E-016	19913121
*PVRL2*	5819	Total cholesterol	1.12E-008	19913121
*CETP*	1071	HDL cholesterol	2.55E-017	18193044
*APOA5*	116519	Triglycerides	2.32E-013	19913121
*ZNF259*	428256	Triglycerides	8.56E-012	19913121
*BUD13*	84811	Triglycerides	5.40E-011	19913121
*HRG*	3273	APTT	2.34E-012	23188048
*HRG*	3273	APC/APTT	2.56E-012	23188048
*HFE*	3077	Haemoglobin	9.76E-012	19862010
*ESR1*	2099	Triglycerides	1.84E-011	16099331
*KNG*	3827	APTT	1.84E-011	20303064
*TFR2*	7036	Haemoglobin	1.04E-009	19862010
*SLC19A2*	10560	APC/APTT	2.21E-009	23188048
*APOC4*	346	LDL cholesterol	8.17E-009	23119086
*NSF*	4905	Triglycerides	2.18E-008	Not Reported
*ACSS2*	55902	Factor VII	2.18E-008	20231535
*EPO*	2056	Haemoglobin	2.31E-008	19862010
*MYBPHL*	343263	LDL cholesterol	3.19E-008	Not Reported
*ACSM3*	6296	Cornell product	2.38E-008	Not Reported
*ERI2*	112479	Cornell product	7.87E-009	Not Reported
*IL18RAP*	8807	Cornell product	1.07E-008	Not Reported
*IL18RAP*	8807	QRS vol. product	1.72E-010	Not Reported
*IL23R*	149233	Cornell product	3.30E-012	23108651
*NRG1*	3084	Cornell product	2.71E-014	Not Reported
*NRG1*	3084	QRS vol. product	2.97E-011	Not Reported

This table shows single phenotype/single genotype association gene-centered association value. The first column represents genes, the second column phenotypes, the third column represents the CCA association value and the fourth column is the Pubmed ID if the association has been previously reported.

Although most genes have more than one associated SNP reading, we found a non-reported association (p = 4.93×10^−10^) between the single SNP rs11264341, located in the intronic region of gene *TRIM46* (ENTREZ GENE # 80128), and the serum magnesium phenotype. This SNP is in LD with SNP rs4072037 (r^2^ = 0.54) in *MUC1*, which has been previously related with serum magnesium. Close to this SNP (>8 kb), and not in LD, we found an association in gene *MUC1* (ENTREZ GENE # 4582) with serum magnesium (p = 1.37×10^−14^) which has been previously reported by Meyer *et al.* [31].

We found a previously reported association of gene *SURF4* (ENTREZ GENE # 6836) and Von Willebrand Factor (vWF), but also a non-previously-reported association with Factor VIII (1.57×10^−24^) and alkaline phosphatase (ALP) (3.1×10^−9^). SNPs in *SURF4* are in some LD (r^2^ = 0.696) with SNP in *C9orf96*, which has been related with vWF [32], also with SNPs in *ABO*(ENTREZ GENE # 28) (r^2^ = 0.502) which has been related with vWF [32] and ALP [33] [34]. We could expect the association between Factor VIII and vWF, because there is a high correlation between its serum concentrations (0.70), and vWF acts as a carrier protein of Factor VIII. However, the correlation between ALP and vWF are 0.14. This is a clear example of vertical pleiotropy, where variants in SURF4 are causal of vWF, and vWF glycoprotein is the carrier for Factor VIII glycoprotein in blood. Another important association (p = 2.18×10^−8^), which has not been reported, is between gene *NSF* (ENTREZ GENE # 4905) and triglycerides. *NSF* is related with genes *KIAA1377* (ENTREZ GENE # 57562) and *LUC7L2* (ENTREZ GENE # 51631), through the PPI network, which are also related with the *LPL* gene (ENTREZ GENE # 4023). Finally the *MYBPHL* gene is associated (p = 3.19×10^−8^) with low density lipoprotein (LDL) cholesterol, which has not been previously reported. However, SNPs in this gene are in LD with SNPs in *CELSR2* (ENTREZ GENE # 1952) (r^2^ = 0.546), *PSRC1* (ENTREZ GENE # 84722) (r^2^ = 1) and *SORT1* (ENTREZ GENE # 6272) (r^2^ 1) rs12740374, which is associated [35], [36], [37], [38], [39] with LDL cholesterol.

### Novel associations of genes with ECG left ventricular hypertrophy

Left ventricular hypertrophy can be detected through ECG parameters such as Cornell product [40] or QRS product [40], [41]. Using the CCA gene-centered association approach we have identified a number of genes associated with these two clinical parameters, which are also positively associated with cardiovascular diseases such as stroke [42]. We found association between *ACSM3* (ENTREZ GENE # 6296) and Cornell product (p = 2.38×10^−8^). This gene was previously reported to associate with hypertension in rats [43] and in humans [44] and also with obesity hypertension in humans [45], but there is some controversy [46]. Other studies relate it with ventricular deformations such as left ventricular mass index and mean wall thickness [47]. The *ERI2* gene was also associated with Cornell product (p = 7.87×10^−9^). This gene overlaps *ACSM3* (*ERI2* SNPs is a subset of *ACSM3*). No association with left ventricular hypertrophy or hypertension has been reported previously. *IL18RAP* (ENTREZ GENE # 8807) was associated with Cornell product (p-value 1.07×10^−8^) and QRS voltage product (p-value 1.72×10^−10^). SNPs in this gene have been associated [48] with echocardiography left ventricular obtained measures. In Grisoni *et al.* [49], using different SNPs in the same gene, the authors did not find any association between *IL18RAP* and any cardiovascular diseases (CVD) risks. However, Tiret *et al.* [50] found a significant association between *IL18* family gene SNPs and mortality. We found association between *IL23R* (ENTREZ GENE # 149233) and Cornell product (3.3×10^−12^). This gene has been associated with left ventricular hypertrophy [51] and idiopathic dilated cardiomyopathy in Chen *et al.* [52]. It is interesting to note the importance of autoimmune related genes (*IL18RAP* and *IL13R*) in left ventricular hypertrophy or idiopathic dilated cardiomyopathy. A relation between autoimmune response and idiopathic dilated cardiomyopathy has been suggested in San Martin *et al.* [53] and Lappe *et al.* [54].

Finally, gene *NRG1* (ENTREZ GENE # 3084) presents an association with phenotypes Cornell product (2.71×10^−14^) and QRS voltage product (2.97×10^−11^). This gene has been associated to cardiovascular development in mouse [55], through the *NRG1*/*ErbB* signaling pathway [56], [57], that is involved in angiogenesis, blood pressure and skeletal muscle response to exercise. In humans, serum NRG-beta has been found elevated in patients with severe systolic heart failure [58]. In McBride *et al.* [59], no association was found between SNPs in *NRG1* and a group of congenital heart malformations (left ventricular outflow tract, defects of aortic valve stenosis, coarctation of the aorta and hypoplastic left syndrome). One of the drawbacks of CCA analysis, which could affect our understanding of the role of *NRG1*, is that this method lacks power when a gene is larger than 100 Kb [27], and *NRG1* has a length of 1.1 Mb.

### Single gene/multiple phenotype analysis

In order to analyze the association of all the SNPs in one gene and multiple phenotypes, we use CCA and a genetic algorithm as an optimization method, to select the most important phenotypes, as described in the Methods section.

**Figure 2:**
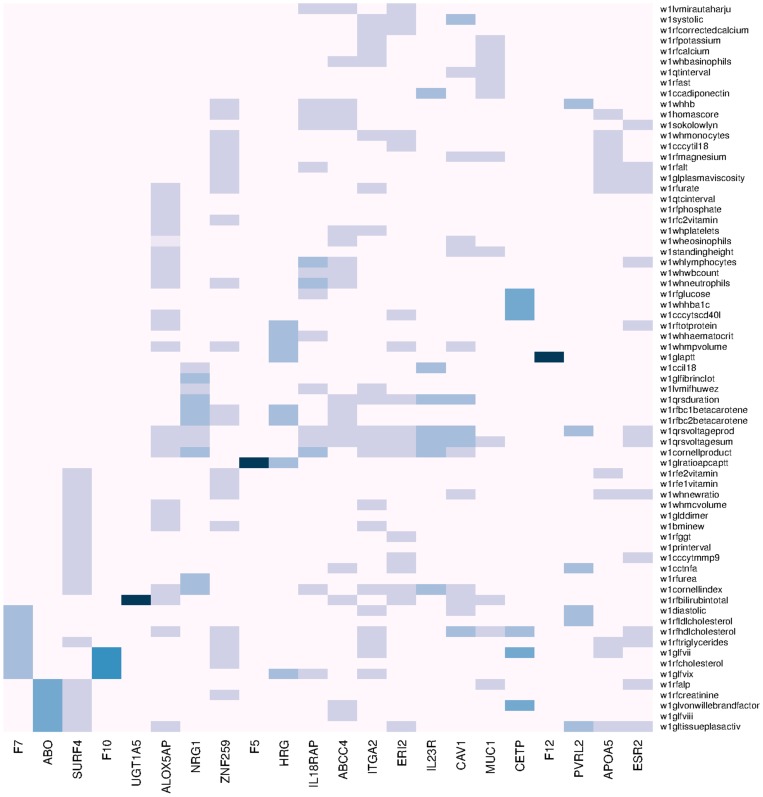
Heatmap single gene/multiple phenotype. This figure shows the 22 most important association rules as columns in a heatmap. Note that only one member of the *UGT1A* family is listed and rules for the same gene are summarized in the same column.

**Figure 3:**
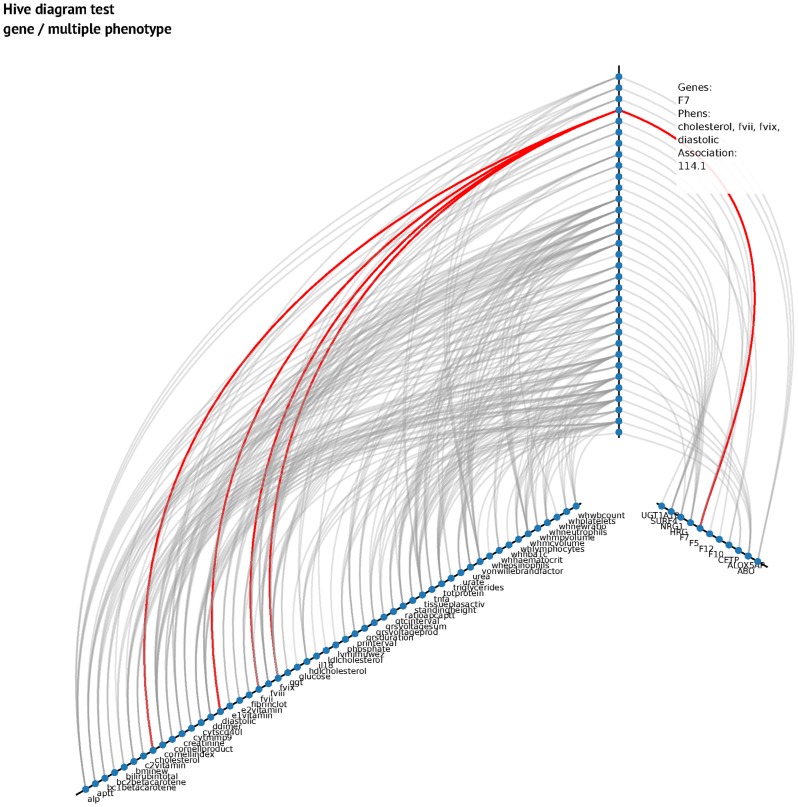
Hive plot single gene/multiple phenotype. This figure shows a hive plot for gene/phenotype association rules. The vertical axis represents the association rules (higher, more association). The left axis represents phenotypes and the right axis represents genes. An interactive hive plot is published on the project webpage (http://pleioexp.epi.bris.ac.uk/cca/geneNphenHive.html).

In Table 2 we show some of the most important pleiotropic genotype/multiple phenotype associations, including the p-value of CCA association and the phenotypes with which they are associated. We also show Fisher's combined association value and, in parentheses, the association value of the genes and the single phenotype. In Table S2 we show all the results for associations between one gene/multiple phenotypes. In order to correct for multiple associations, we use a Bonferroni correction for 3648 genes and combinations of 82 phenotypes in subsets of 24 to 2 groups. We chose 24 because it is the maximum number of different phenotypes in one association rule (an association rule is a combination of a number of phenotypes associated with a number of genes) selected by the genetic algorithm (see the multiple test association correction paragraph in Methods). This combination gives 5.36×10^20^ different phenotypic rules, giving a threshold p-value of 2.55×10^−25^ equivalent to p = 0.05 for a single test. In Figure 2, we use a heatmap plot to represent the most important (higher association) pleiotropic relations between phenotypes and genotypes. Also, we use a hive plot (interactive plot available online) in Figure 3. In this diagram, vertical axis represents the association between the phenotype (left axis) and genotype (right axis). Association rules are ordered in the diagram following the association value (the higher association, the higher in the plot).

**Table 2:** Single gene/multiple phenotype association.

Gene	Entrez gene#	CCA p-value	Fisher p-value	Phenotypes
*ABO*	28	3.47E-147	1.53E-194	Creatinine (0.2771) ALP (4.35e-12) FVIII (2.288e-77) Tissue plasminogen activator (0.002849) Von Willebrand Factor (9.43e-112)
*F7*	2155	4.42E-114	7.27E-078	Cholesterol (0.9719) HDL cholesterol (0.1073) FVII (1.486e-79) FIX (0.5965)
*F10*	2159	3.58E-079	1.61E-059	Cholesterol (0.8131) FVII (1.24e-60) FIX (0.8744)
*SURF4*	6836	2.02E-056	1.98E-066	Mean cell volume (0.01924) ALP (3.104e-09) GGT (0.7735) E vitamin_t1 (t1) (0.1303) E vitamin_t2 (0.03584) FVIII (1.573e-24) D-dimer (0.4159) Tissue plasminogen activator (0.1705) Von Willebrand Factor (5.655e-37) MMP9 (0.006049) BMI (0.1158) PR interval (0.04652) Cornell index (0.001895)
*NRG1*	3084	1.03E-028	4.73E-019	Urea (0.005643) beta-carotene (0.001871) fibrinogen (0.007379) QRS duration (0.0008578) Cornell index (0.651) Cornell product (2.709e-14)
*HRG*	3273	8.02E-024	1.03E-022	Haematocrit (0.05769) Total protein (0.007418) FIX (0.3289) APTT (2.342e-12) Ratio APC/APTT (2.555e-12)
*IL18 RAP*	8807	5.07E-021	1.52E-017	White blood count (0.1395) Haematocrit (0.04132) Neutrophils (0.4115) Lymphocytes (0.831) ALT (0.0287) Glucose (0.06899) FIX (0.03512) Cornell index (0.4867) Cornell product (1.072e-08) Sokolowlyn index (0.2884) QRS voltage product (1.723e-10)
*CETP*	1071	3.65E-020	5.94E-020	HBA1C (0.01231) HDL cholesterol (2.552e-17) Glucose (0.06243) FVII (0.0178) Von Willebrand Factor (0.003123) CD40 (0.01286)
*ALOX5AP*	241	3.49E-018	7.28E-011	White blood count (0.2124) Mean cell volume (0.004036) Neutrophils (0.007984) Urate (0.02255) Phosphate (0.07024) Total protein (0.002632) C Vitamin (0.06383) Tissue plasminogen activator (0.06189) CD40 (0.02214) BMI (0.03787) Height (0.002794) QTC interval (0.08854) Cornell index (0.1151) QRS voltage sum (0.2935) QRS voltage prod (0.08101)
*IL23R*	149233	2.19E-018	1.09E-012	Adiponectin (0.04484) IL18 (0.01227) QRS duration (0.1137) Cornell index (0.2268) Cornell product (3.299e-12)
*GPR98*	84059	3.37E-008	1.31E-006	White blood count (0.005413) Mean platelet volume (0.1353) Lymphocytes (0.2363) Potassium (0.08101) Sodium (0.05957) Total protein (0.02314) E vitamin (0.03851) Insulin (0.05201) Adiponectin (0.1668) TNFA (0.2093) homa-score (0.2542) CVD (0.02455)

This table shows single gene/multiple phenotype association. The first column represents the gene, the second column represents the CCA association value, the third column is the Fisher's combined association value and the fourth column the phenotypes associated. In parentheses is the single phenotype association value of each phenotype.

Gene *ABO* which has an indicated association (p-value 2.47×10^−147^) with coagulation (tissue plasminogen activation, Factor VIII and Von Willebrand factor levels), but also with serum levels of ALP (previously reported in Yuan *et al.* [34] and creatinine. Gene *SURF4* (ENTREZ GENE # 6836), which has been previously associated with Von Willebrand Factor, Factor VIII and ALP, is also associated with ECG measures, MMP-9 (inflammatory marker) and mean cell volume (average red blood cell volume) among others. Gene *HRG* presents a weak association (p-value 1.88×10^−24^, corrected threshold 2.55×10^−25^) with some factors related with coagulation, such as activated partial thromboplastin time (APTT), ratio activated protein C (APC)/APTT, volume, total protein and Factor IX. Finally gene *CETP* (ENTREZ GENE # 1071) shows weak association (p-value 3.65×10^−20^) with cholesterol as expected, but also with coagulation factors (Von Willebrand Factor, Factor VII and sCD40 ligand).

Gene *F10* (ENTREZ GENE # 2159), presents association with coagulation factors (Factor VII and Factor IX), but also with cholesterol, similar to gene *F7*, which also presents association with diastolic blood pressure. For the gene *NRG1*, we found association with ECG measures of ventricular hypertrophy, but also with urea and fibrinogen. Gene *IL18RAP* is weakly associated (p-value 5.07×10^−21^) with white cell counts (white cells, neutrophils, lymphocytes), with alanine transaminase (ALT) and glucose, but also with ECG measures of ventricular hypertrophy. Gene *IL23R* is weakly associated (p- value 2.19×10^−18^) with levels of interleukin 18 but also with adiponectin and ECG measures of ventricular hypertrophy). Gene *ALOX5AP* (ENTREZ GENE # 241) has been related with myocardial infarction and stroke [60], and also with inflammatory activity and atherosclerosis [61]. In our results it presents some association with some phenotypes related with immune response (white blood count, neutrophils, CD40 or total protein) but also with some markers of ECG related with hypertension. And gene *GPR98* (ENTREZ GENE # 84059) is related in our analysis with immune response phenotypes and insulin related phenotypes (insulin, HOMA score) and in some cases with an association with CVD. No relation between this gene and these phenotypes has been reported, but some association was reported with carotid diseases and body weight [62], [63].

### Multiple gene/single phenotype analysis

In this case, instead of selecting the most associated phenotypes for each gene, the GA selects the most associated genes for each phenotype. This operation is more computationally expensive than the previous one, because of the high number of genes (3648) involved. In order to correct for multiple testing, we use a Bonferroni correction for 82 phenotypes and a combination of 3648 genes in subsets of 29, 28, 27…1 groups. We choose 29 because this is the maximum number of different genes in one rule. This combination gives 2.03×10^72^ different genotypic rules, giving a threshold p-value of 2.99×10^−75^ equivalent to p = 0.05 for a single test. Ferreira [27] comments there may be a lack of power related with gene size for CCA for the case of multiple gene analysis. However, we consider that this analysis could contribute if the involved genes are small. Some of the most interesting rules are shown in Table 3. Other significant and non-significant enrichment analyses of the genes in the rules are listed in supplementary Table S3 and S4.

**Table 3:** Multiple gene/single phenotype association table.

Phenotype	CCA p-value	Fisher p-value	Genes
Von Willebrand Factor	2.48E-119	3.12E-115	*RTN4RL1 (0.08536) ABO (9.43e-112) HOXA7 (0.001082) MED1 (0.001762) RPS6KA2 (0.001278) PDIA2 (0.004177) TCEA3 (0.03156) PPP1R1B (0.0007463)*
Bilirubin total	3.79E-118	7.80E-113	*SGSM1 (0.00048) EIF2C1 (0.06558) IFNG (0.1578) ODF3B (0.0004058) UGT1A8 (1.293e-112) TSC1 (0.04822) LRBA (0.1041)*
Factor VII	8.49E-087	3.09E-083	*EMILIN1 (0.0762) F7 (1.486e-79) SERPINC1 (0.0126) EDEM2 (3.111e-07) PRTFDC1 (0.01879)*
Factor VIII	7.01E-082	1.28E-077	*CEBPB (0.01432) CSMD2 (0.0168) ABO (2.288e-77) RELN (0.06236) TNR (0.02139) C11orf9 (0.002082) NSMAF (0.09858) TRIM55 (0.02656) TXNL1 (0.0164)*

This table shows multiple gene/single phenotype association values. The first column is the phenotype, the second column is the CCA association value, the third column is Fisher's combined association value and the fourth column are the genes associated. In parentheses the single gene/single phenotype association value is given for each gene.

The Von Willebrand factor association (p-value 1.69×10^−117^–2.49×10^−119^) is led by individual association with gene *ABO* (p-value 9.43×10^−112^), and two of three significant pathways present more CCA association that Fisher multiple association.

The bilirubin association (p-value 6.76×10^−115^–3.79×10^−118^) is most influenced by genes in the *UGT1* family, and all pathways present more CCA association than Fisher multiple association. The FVII association is led by genes *F7* and *EDEM2* (ENTREZ GENE # 55741) or *PROCR* (ENTREZ GENE # 10544). Finally FVIII association is led by *ABO* gene.

Regarding the enrichment analysis (Table S4), some interesting enrichments has been found, such as Factor VII and Human Phenotype Pathway “Abnormality of the coagulation cascade”, KEGG pathway “Complement and coagulation cascades” and Reactome pathway “Formation of Fibrin Clot (Clotting Cascade)”, or Factor VIII and KEGG pathways “ECM-receptor interaction” (pathway related with hemophilia, directly related with factor VIII). From non significant rules, APTT related genes are annotated with GO Terms “negative regulation of blood coagulation”, “blood coagulation fibrin clot formation”, “blood coagulation intrinsic pathway” and Reactome pathway “formation of fibrin clot”. Finally, LDL cholesterol is annotated with *LDL* gene related annotations

### Multiple gene/multiple phenotype analysis

Finally, we use a CCA - two population genetic algorithm approach for multiple gene/multiple phenotype rule extraction. As a result, a set of 56 rules that relate the most associated set of genes with phenotypes was obtained. Following our previous multiple association corrections, the maximum number of genes in the obtained rules is 22 and the maximum size of the phenotypes is 9, so there is a possible population of 1.94×10^57^ gene rules and 3.3×10^11^ phenotypes, that determine a threshold p-value of 7.71×10^−70^ (equivalent to p = 0.05 for a single test).

Table 4 shows some of these association rules, and a complete list of 56 rules can be found in Table S5. An enrichment analysis can also be found in Table S6.

**Table 4:** Multiple gene/multiple phenotype association table.

Genes	CCA p-value	Phenotypes
*F7*, *ABO*, *MRPS28*, *UGT1A3*, *SURF4*	<2.22E-308	Bilirubin, Factor VII, Von Willebrand Factor
*F5*, *ABO*, *MYO1B*, *PAX5*, *TRIM46*	1.35E-168	ALP, Ratio APC/APTT, Von Willebrand Factor
*F5*, *UGT1A3*	6.68E-153	Bilirubin, Ratio APC/APTT
*F7*, *EDEM2*	1.56E-123	Cholesterol, factor VII, factor IX, diastolic blood pressure

This table shows multiple gene/multiple phenotypes association rules. The first column are the multiple genes, the second column the CCA association value and the third column the multiple phenotypes.

The bigger association obtained rule, genes *F7*, *ABO*, *MRPS28*, *UGT1A3 and SURF4* with phenotypes bilirubin FVII and vWF, presents an association probability under 2.22×10^−308^, which was below our machine precision and therefore recorded as zero. We have identified some patterns in the multiple genes/multiple phenotype pleiotropic rules. *ABO* and *SURF4* has similar relations with ALP, FVIII and vWF, *F7* and *F5* with FVII, *F5* and *HRG* with APTT and ratio APC/APTT, *F12* with APTT and *NRG* with Cornell product and QRS voltage product. Most of the rules obtained here are combinations of these.

The enrichments analysis of multiple phenotypes reveals interesting results, such as a rule formed by phenotypes bilirubin, alp, APTT, ratio APC/APTT and Von Willebrand Factor which were enriched for HP pathways “Prolonged partial thromboplastin time” and “Prolonged whole-blood clotting time”, KEGG pathway “Complement and coagulation cascades” and Reactome pathway “Formation of Fibrin Clot (Clotting Cascade)”. This rule is not a clear example of pleiotropy, because all genes and phenotypes are related with clotting, but it is clear that the inclusion of all genes and phenotypes in the same rules increases the association. Rules including phenotypes QRS duration, Cornell Index and Cornell Product, are annotated with hypertension GO terms and linked with genes that support these annotations. Also rules including phenotypes related with left ventricular hypertrophy are enriched with the GO term “epithelium development” and linked with genes related with cardiovascular development.

## Discussion

In the case of single gene/single phenotype analysis, we are not looking for pleiotropic effects, but for a combined gene-based association effect, and some interesting results were found. The complete list of gene-based significant and previously reported associations can be found in Table 1. One of the drawbacks of CCA analysis, which could affect our understanding of the role of *NRG1*, is that this method lacks power when a gene is larger than 100 Kb [27], and *NRG1* has a length of 1.1 Mb.

In the case of single gene and multiple phenotype association, our results show that the p-values (both CCA and Fisher) increase when more related phenotypes are included in the phenotype set. As expected, most of these phenotypes are correlated/associated. However, not all phenotypic sets are correlated. An example can be observed in gene *F7* (ENTREZ GENE # 2155), which is associated with phenotypes total cholesterol, Factor VII and Factor IX. Correlation exists between total cholesterol and Factor VII (0.28), Factor VII and Factor IX (0.39), but not between cholesterol and Factor IX (0.09). In some cases, Fisher's combined p-value approach shows equal or bigger association than CCA, which could mean that CCA association shows the cumulative effects of individual associations. In contrast, when Fisher's multiple association p-value is smaller than CCA association, this could suggest that CCA association analysis has found pleiotropic effect between a gene and these phenotypes. Some examples of the first group are association of gene *ABO* with coagulation phenotypes. In contrast, examples of pleiotropic effects appear in genes *F10* (ENTREZ GENE # 2159) or F7, which presents an association with coagulation factors (Factor VII and Factor IX), but also with cholesterol.

In the case of multiple gene/single phenotype, using this robust association threshold, we have identified a set of pathways that are associated significantly with phenotypes Von Willebrand factor, bilirubin, FVII and FVIII. The whole list of pathways is listed in Table S3. It's interesting to see that there is no significant difference between the CCA and Fisher's association, in contrast with the differences shown in the previous Section, which supports the fact that CCA could detect pleiotropy patterns.

In conclusion, in this paper we have applied a canonical correlation analysis approach for association in multivariate datasets, finding correlations between gene-centered genetic variants and phenotypes. This multivariate approach allows us to mine pleiotropic relations between one or a set of genes and a set of phenotypes. In term of single gene/single phenotype association, we have found non-reported associations of gene NSF and triglycerides and genes *ACSM3*. *ERI2*, *IL18RAP*, *IL23RAP* and *NRG1* with phenotypes related with left ventricular hypertrophy. We use a genetic algorithm as feature selection algorithm in order to find pleiotropy patterns in phenotypes. Using this approach we found pleiotropy patterns in genes *F7* and *F10* with phenotypes Factor VII, Factor IX and cholesterol; *NRG1*, with left ventricular hypertrophy related phenotypes, but also with fibrinogen and urea or IL18RAP or IL23RAP, related with immune response related phenotypes, but also with ECG measures.

Despite the possible drawbacks of CCA, related to power when the length of a gene is greater than 100 Kb, or increases of type I error when features are not normally distributed, we found that CCA can be used as a powerful tool to find gene-centered association, multivariate association and pleiotropic patterns. Also, this tool can be extended to find non-linear canonical correlation relations using kernel based approaches such as KCCA. Future research directions include improving the search method, using other meta-heuristics such as Tabu Search, Simulated Annealing or Particle Swarm Optimization, or sparse regularization methods.

## Materials and Methods

### The study population

The British Women's Heart and Health Study (BWHHS) is a UK-based prospective cohort study of 4286 healthy women aged 60–79 years at baseline (1999–2001). Participants were selected at random from general practice registers in 23 UK towns [64]. A range of baseline data sources (blood samples, anthropometry, health/medical history, echocardiography measures, etc.) was collected between 1999 and 2001, and DNA extracted from 3884 participants. Although the cohort has been followed-up in subsequent phases, all data presented here is based on the recruitment (baseline) phase.

### Ethics statement

Multi-centre (London Multi-centre Regional Ethics Committee) and local research ethics committees provided approval for the BWHHS study and informed consent was obtained from the women to complete the data used in this study.

### Genotyping

Genotyping was performed using the Illumina HumanCVD BeadArray (Illumina Inc, San Diego, USA), which comprises nearly 48,742 SNPs in over 2,100 genes selected on the basis of cardiovascular candidacy by an international consortium of experts [65]. Genotypes were called using a Illumina BeadStudio (v3) Genotyping Module. Samples with a genotype call rate <90%, Hardy Weinberg disequilibrium <10^−7^ and minor allele frequency <1% were excluded from the analysis, following insight from previous work on this array and patient cohort [66]. Non-European samples were also excluded from analysis. Principal components analysis identified no evidence of population stratification (consistent with self-reported ancestry).

### Phenotyping

The different phenotypes used in this study consisted of 11 directed and derived electrocardiogram (ECG) measures, obtained as described in Gaunt *et al.* [67], 64 blood measures, 2 blood pressure readings, 3 anthropometric measures, HOMA score (derived from glucose and insulin values) and an indicator of whether a patient has suffered cardiovascular disease. These data were measured as described in Lawlor *et al.* [64].

### Data preprocessing

All data were analyzed using R (The R project for statistical computing, http://www.r-project.org/). Due to the high number of missing values present in the phenotypic data (7575 of 312984 values, median of 55 (1.42%) missing values per phenotype, max 509 (13.17%) and min 19 (0.49%)), we followed a strategy of phenotypic data imputation based on a *k*-nearest neighbor approach, implemented in the R package “Imputation” [68] (http://cran.r-project.org/web/packages/imputation/index.html), with a k of 5. In order to test how these imputed values affected the association profile, we compared the single association values of imputed data versus data with missing values removed. The results show that the associations are the same or lower in the imputed values, so imputation does not create false associations. All phenotypic data was normalized to mean zero and standard deviation one.

All the approaches for analysis in this work were based on a “gene-centered” perspective. Genotype data, both intronic and exonic, was assigned to the genomically closest gene using the function “ClosestBED” from the suite “BEDTools” [69] (http://bedtools.readthedocs.org/en/latest/). In order to avoid multicollinearity in genotype data, we applied two-stage linkage disequilibrium (LD) pruning as described in Tang and Ferreira [27]. We removed SNPs with a high LD (r^2^>0.64) with other markers and also a high correlation between linear combinations of SNPs using Variance Inflation Factor (VIF) [70] in order to exclude SNPs with a VIF>2 with other markers. In order to select the most appropriate value for r^2^, we developed several experiments to test the CCA single gene/single phenotype association using a range of r^2^ (0.5–0.99), and best results was obtained pruning SNPs with r^2^>0.64. The value of VIF>2 was selected based in the recommendation of the original CCA paper [27].

### The study design

As mentioned above, unlike other approaches to pleiotropy analysis, in this study we used a gene-centered approach. This perspective allowed us to capture all the pleiotropic effects in one gene, instead of the pleiotropic effects caused by just one variation. But we are also interested in studying the pleiotropic effects of a set of genes in several phenotypes. In order to do this, we divided the study into four stages. Firstly, we studied the individual association between each gene (which may consist of one or more SNPs) and a single phenotype to establish a gene-centered association baseline. This approach did not reveal any pleiotropy, of course, but it is worth pursuing for two reasons. Firstly, it was interesting to find if inclusion of several SNPs increases the association value over a single SNP approach. Secondly, we got a baseline gene association value that we used as a comparator for the CCA association analyses in our subsequent analysis.

For our second stage we studied the association between one single gene and a set of phenotypes. The aim of this analysis was to reveal possible gene-based pleiotropic effects. Our next stage was to study association effects between multiple genes and a single phenotype (gene-based epistasis analysis). The aim of this analysis was to discover pathway based baseline association between a set of genes and a single phenotype. Finally, our last stage consisted in studying the association between a set of multiple genes and different phenotypes. Here we expected to find the pleiotropic effects of a set of genes in multiple phenotypes, with increased statistical significance for the indicated association rules.

### Canonical Correlation Analysis

Canonical Correlation Analysis (CCA) allows us to find linear combinations of two sets of variables with the highest correlations. The aim of this work was to find correlation between a set of genotype data and a set of phenotype data. The CCA algorithm was based on a method proposed by Tang and Ferreira [27]. In order to test the significance of all canonical correlations, Wilk's Lambda and Rao's F approximation were calculated. Let *q* be the number of SNPs in the genotype, *p* the number of phenotypes evaluated, *n* the number of samples and *c*_j_ the number of canonical components calculated. Wilk's Lambda is calculated as follow:
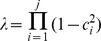
And Rao's F approximation:
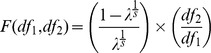
Where
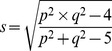


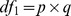


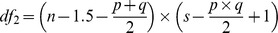
The methods for CCA analysis analyzed in the previous Section could be computationally expensive with a large number of features and samples. In order to use standard CCA we can also divide the feature set into small subsets using biological insight (eg the set of SNPs in the region of a specific gene). In this paper we will simply use feature sets in which the SNPs are linked to a single gene, defining the link to a gene by genomic proximity. In this case, feature selection is not necessary because the number of samples is larger than the number of features.

To find those sets which have a high correlation, according to CCA, we need to use an optimization method, with the association value as the fitness function for this optimization procedure. We have formulated this optimization step as an integer programming problem which can therefore be addressed using a metaheuristic procedure to find an approximately good solution in a computationally tractable time. We have decided not to use methods such as hill climbing or similar local methods, because they are prone to capture by local minima. In this particular problem, any big single association could be assumed to be a local minimum and a hill climbing approach could not exit easily. Instead of this, we have decided to use global methods, such as Tabu Search [71], Particle Swarm Optimization [72] or Genetic Algorithm (GA) used here, as a well known and well used approach to this type of problem and with an effective means for evading local minima. A GA is a metaheuristic, initially proposed by Holland [73] and Goldberg [74]. This procedure is based on the principles of evolution and natural selection, with steps analogous to inheritance, mutation and crossover. It is initialized with a set of solutions, each representing one possible solution to the problem. The performance of each proposed solution is estimated using the fitness function, which measures how well an individual solution is adapted to the proposed problem. The method then iteratively evolves a high-fitness solution. The “genalg” R package (http://cran.r-project.org/web/packages/genalg/index.html) was used as a binary implementation of a GA. However, because of the requisites of the multiple gene/multiple phenotype analysis, this code was modified in order to include two population searches (Modified source of genealg package is available in http://github.com/jseoane/gaCCA). One of those populations represents different solutions for gene selection, and the other represents different solutions for phenotype selection. The search strategy is applied in parallel over the two populations and the fitness function is evaluated simultaneously over the selected set of genes and selected set of phenotypes, when calculating their CCA association value.

The encoding of the genetic algorithm is a binary encoding, widely used in feature selection approaches, where if the feature is set to 1, it is included in the analysis and is not included otherwise. The fitness function in the three versions is defined by the CCA association value. Regarding the parameterization, the population size is 100 for single gene/multiple phenotype, 600 for multiple gene/single phenotype and 1000 for multiple gene/multiple phenotype. The mutation depends on the size of the GA chromosome (1/82 in the case of multiple phenotype, 1/3248 for multiple gene). The elitism (how many samples of the population are conserved between generations) is 20/35/100, respectively for each of the versions. Finally, the “zero to one ratio”, which controls the number of features in the chromosome is set to 50 in the case of multiple phenotype and 700 in the case of multiple gene.

In order to avoid multiple testing associations which arise by chance, we applied a Bonferroni correction. In this case, the Bonferroni correction should be applied to both sides of the association. In this case the association is calculated over *p* phenotypes and *g* genes, so a 0.05 of confidence should need 0.05/(p*g). But when a GA is used, millions of associations are considered, so we approximate the Bonferroni correction over the search space of the algorithm (i.e. if we expect rules of *p*′ phenotypes and *g*′ genes, the search space over genes are combinations of *g* different genes over *g*′, *g′*-1, *g*′-2,‥,2, and the search space over phenotypes are combinations of *p* different phenotypes over *p*′, *p*′-1, *p*′-2,‥,2). The highly conservative final association threshold proposed is 0.05/(length of search space in genes * length of search space in phenotypes), though we ranked and considered all results by p-value in our analysis. In order to compare the CCA combined association measure with other measures, we have chosen a statistical measure based on Fisher's combined p-value approach proposed in Li *et al.* [5].

### Enrichment analysis

During phase two and phase four of the analysis, a set of genes related with one or several phenotypes is obtained. In order to functionally annotate these sets of genes, we perform an enrichment analysis, detecting GO ontology terms, KEGG, Reactome or Phenotype annotations that are significantly present in our pathways. We use the enrichment analysis tool g:Profiler [75] (http://biit.cs.ut.ee/gprofiler/), through R package “gProfiler” (http://cran.r-project.org/web/packages/gProfileR/index.html). In order to calculate the p-values for each enrichment, the method first simulate 10 millions of queries (sets of genes) randomly to see how was the p-values distribution according the query size. Then analytically derived the p-value threshold for each query size (for more details consult g:SCS threshold section in the Reimand paper).

### Precision

Because some association values were close to zero, note that all calculations were performed in a 64-bit Linux R environment where the lowest positive value is 2.22×10^−308^, which means that values below this threshold were treated as zero.

## Supporting Information

Table S1 Single gene/single phenotype CCA association values. This table shows the p-values and the minus log 10 association values for single gene/single phenotype CCA analysis.
(DOC)

Table S2 Single gene/multiple phenotype CCA association rules. This table shows the association values in CCA single gene/multiple phenotype analysis. The second column shows the CCA association value, the third column represents the Fisher's combined association value and the fourth column shows the phenotypes associated. In parentheses is the single phenotype association value for each phenotype.
(XLSX)

Table S3 Multiple genes/single phenotype CCA association rules. This table shows the association values in CCA multiple gene/single phenotype analysis. The first column shows the phenotypes, the second column shows the CCA association value, the third column represents the Fisher's combined association value and the fourth columns shows the genes associated. In parentheses is the single gene/single phenotype association value for each gene.
(XLSX)

Table S4 Enrichment analysis multiple gene/single phenotype. This table shows the results of enrichment analysis performed with g:Profiler for each rule obtained in multiple gene/single phenotype analysis. The first column shows the enrichment p-value, the second column shows the term id following the gProfiler terminology. Term starting with GO refers to Gene Ontology, terms starting with BIOGRID refers to Biogrid interaction data, terms starting with TF refers to a Transcriptor Factor from TRANSFAC database, terms starting with KEGG refers to a KEGG database pathway, terms starting with MI refers to a microRNA targets sites from MicroCosm database, terms starting with CORUM refers to CORUM database protein complexes, terms starting with REAC refers to a Reactome database pathway and finally terms starting with HP refers to terms in Human Phenotype Ontology (HPO). The third column is the name of pathway/GO term, etc. The fourth column represents the genes present in the enrichment. The fifth column shows the phenotype and the last column represent the CCA association p-value.
(XLSX)

Table S5 Multiple genes/multiple phenotype CCA association rules. This table shows the association values in CCA multiple gene/multiple phenotype analysis. The first column shows the phenotypes, the second column represents the CCA association value and the thirst column shows the genes associated.
(DOC)

Table S6 Enrichment analysis multiple gene/multiple phenotype. This table shows the results of enrichment analysis performed with g:Profiler for each rule obtained in multiple gene/multiple phenotype analysis. The first column shows the enrichment p-value, the second column shows the term id following the g:Profiler terminology. Term starting with GO refers to Gene Ontology, terms starting with BIOGRID refers to Biogrid interaction data, terms starting with TF refers to a Transcriptor Factor from TRANSFAC database, terms starting with KEGG refers to a KEGG database pathway, terms starting with MI refers to a microRNA targets sites from MicroCosm database, terms starting with CORUM refers to CORUM database protein complexes, terms starting with REAC refers to a Reactome database pathway and finally terms starting with HP refers to terms in Human Phenotype Ontology (HPO). The third column is the name of pathway/GO term, etc. The fourth column represents the genes present in the enrichment. The fifth column shows the phenotypes and the last column represent the CCA association p-value.
(XLSX)

Table S7 Glossary of phenotypes. Complete name of the phenotype.
(DOC)
