# Supplementary material for: Canonical Correlation Analysis for Gene-Based Pleiotropy Discovery
Source: PLoS Comput Biol. 2014 Oct 16;10(10):e1003876. doi: 10.1371/journal.pcbi.1003876 (PMC4199483; doi:10.1371/journal.pcbi.1003876)
Supplement: Table S1 — Single gene/single phenotype CCA association values. This table shows the p-values and the minus log 10 association values for single gene/single phenotype CCA analysis. (DOC) [file pcbi.1003876.s001.doc]

Table S1: Single gene / single phenotype CCA association values

| genename | Pvalue | phenname | log10 |
| --- | --- | --- | --- |
| UGT1A10 | 1.29E-112 | bilirubintotal | 111.8883721692 |
| UGT1A8 | 1.29E-112 | bilirubintotal | 111.8883721692 |
| UGT1A7 | 1.29E-112 | bilirubintotal | 111.8883721692 |
| UGT1A6 | 1.29E-112 | bilirubintotal | 111.8883721692 |
| UGT1A9 | 1.29E-112 | bilirubintotal | 111.8883721692 |
| UGT1A5 | 3.21E-112 | Bilirubintotal | 111.4931153385 |
| UGT1A4 | 3.21E-112 | Bilirubintotal | 111.4931153385 |
| UGT1A3 | 3.21E-112 | Bilirubintotal | 111.4931153385 |
| ABO | 9.43E-112 | vonwillebrandfactor | 111.0254844514 |
| UGT1A1 | 2.56E-110 | Bilirubintotal | 109.5922791297 |
| F7 | 1.49E-079 | Fvii | 78.8281162856 |
| ABO | 2.29E-077 | Fviii | 76.640503967 |
| F5 | 2.77E-062 | Ratioapcaptt | 61.5575892113 |
| F10 | 1.24E-060 | Fvii | 59.9065541776 |
| SURF4 | 5.66E-037 | vonwillebrandfactor | 36.2475351346 |
| F12 | 5.77E-035 | Apt | 34.2387913265 |
| SURF4 | 1.57E-024 | Fviii | 23.8032075018 |
| CETP | 2.55E-017 | hdlcholesterol | 16.5931846032 |
| PVRL2 | 2.26E-016 | ldlcholesterol | 15.6453881789 |
| MUC1 | 1.37E-014 | Magnesium | 13.8634335774 |
| NRG1 | 2.71E-014 | cornellproduct | 13.5672463234 |
| APOA5 | 2.32E-013 | Triglycerides | 12.6349128068 |
| HRG | 2.34E-012 | Apt | 11.6304720496 |
| HRG | 2.56E-012 | Ratioapcaptt | 11.5925404393 |
| IL23R | 3.30E-012 | cornellproduct | 11.4815958056 |
| ABO | 4.35E-012 | Alp | 11.3614945823 |
| ZNF259 | 8.56E-012 | Triglycerides | 11.0674638635 |
| HFE | 9.76E-012 | mchaemoglobin | 11.0107630586 |
| ESR2 | 1.84E-011 | Triglycerides | 10.735092405 |
| NRG1 | 2.97E-011 | qrsvoltageprod | 10.52749728 |
| KNG1 | 5.36E-011 | Apt | 10.2707747635 |
| BUD13 | 5.40E-011 | Triglycerides | 10.2673140504 |
| IL18RAP | 1.72E-010 | qrsvoltageprod | 9.7636781581 |
| TRIM46 | 4.93E-010 | Magnesium | 9.3067916123 |
| TFR2 | 1.04E-009 | mchaemoglobin | 8.9845756425 |
| SLC19A2 | 2.21E-009 | Ratioapcaptt | 8.6563144204 |
| SURF4 | 3.10E-009 | Alp | 8.5081065521 |
| ERI2 | 7.87E-009 | cornellproduct | 8.1042555985 |
| APOC4 | 8.17E-009 | ldlcholesterol | 8.0875323267 |
| IL18RAP | 1.07E-008 | cornellproduct | 7.9696261735 |
| PVRL2 | 1.12E-008 | Cholesterol | 7.9520974181 |
| NSF | 2.18E-008 | Triglycerides | 7.6614038908 |
| ACSS2 | 2.18E-008 | Fvii | 7.6607219317 |
| EPO | 2.31E-008 | mchaemoglobin | 7.6361687291 |
| ACSM3 | 2.38E-008 | cornellproduct | 7.6228505748 |
| MYBPHL | 3.19E-008 | ldlcholesterol | 7.4957775032 |
| CAV1 | 7.15E-008 | cornellproduct | 7.1456750546 |
| ACTL6B | 9.14E-008 | mchaemoglobin | 7.039109022 |
| TOMM40 | 1.03E-007 | ldlcholesterol | 6.9855438643 |
| IL16 | 1.49E-007 | cornellproduct | 6.8272452461 |
| USF1 | 2.20E-007 | cornellproduct | 6.6575357452 |
| IL23R | 2.39E-007 | qrsvoltageprod | 6.6220580245 |
| ABCC4 | 2.76E-007 | qrsvoltageprod | 6.5590201376 |
| EDEM2 | 3.11E-007 | Fvii | 6.5071219561 |
| CRP | 3.17E-007 | creactiveprotein | 6.4990870123 |
| TFR2 | 4.45E-007 | Rbcount | 6.3513098181 |
| HFE | 5.76E-007 | Hb | 6.2392833007 |
| ITGA2 | 6.71E-007 | cornellproduct | 6.173264929 |
| ITGA4 | 9.06E-007 | Monocytes | 6.0427812209 |
| USF1 | 1.06E-006 | qrsvoltageprod | 5.9759670536 |
| TBC1D1 | 1.33E-006 | Tnfa | 5.8774637404 |
| F12 | 1.47E-006 | Fvix | 5.8315435268 |

Legend: This table shows the p-values and the minus log 10 association values for single gene / single phenotype CCA analysis.
