# Supplementary material for: Canonical Correlation Analysis for Gene-Based Pleiotropy Discovery
Source: PLoS Comput Biol. 2014 Oct 16;10(10):e1003876. doi: 10.1371/journal.pcbi.1003876 (PMC4199483; doi:10.1371/journal.pcbi.1003876)
Supplement: Table S5 — Multiple genes/multiple phenotype CCA association rules. This table shows the association values in CCA multiple gene/multiple phenotype analysis. The first column shows the phenotypes, the second column represents the CCA association value and the thirst column shows the genes associated. (DOC) [file pcbi.1003876.s005.doc]

Table S5: Multiple genes / multiple phenotype CCA association rules

| Genes | CCA association p-value | Phenotypes |
| --- | --- | --- |
| F7 ABO MRPS28 UGT1A3 SURF4 | 0 | bilirubintotal fvii vonwillebrandfactor |
| F10 ABO UGT1A9 | 2.73E-288 | bilirubintotal alp fvii fviii fvix tissueplasactiv vonwillebrandfactor |
| F7 ABO ACSS2 | 1.15E-246 | alp cholesterol fvii fviii fvix tissueplasactiv vonwillebrandfactor |
| ABO UGT1A5 TRIM46 | 3.35E-245 | magnesium bilirubintotal alp fviii tissueplasactiv vonwillebrandfactor |
| F7 UGT1A8 SURF4 | 3.66E-242 | bilirubintotal alp cholesterol fvii fvix tissueplasactiv vonwillebrandfactor |
| ABO UGT1A5 | 4.93E-242 | bilirubintotal alp fviii tissueplasactiv vonwillebrandfactor |
| ABO UGT1A3 | 4.93E-242 | bilirubintotal alp fviii tissueplasactiv vonwillebrandfactor |
| F5 F12 UGT1A1 SURF4 | 4.22E-189 | bilirubintotal alp aptt ratioapcaptt vonwillebrandfactor |
| F5 ABO MYO1B PAX5 TRIM46 | 1.35E-168 | alp ratioapcaptt vonwillebrandfactor |
| F5 PPPDE2 ABO MAPK3 | 1.32E-165 | alp ratioapcaptt vonwillebrandfactor |
| LTB4R F5 MYH1 UGT1A5 TIA1 TEAD2 RNF8 | 8.77E-155 | bilirubintotal ratioapcaptt |
| F5 UGT1A3 | 6.68E-153 | bilirubintotal ratioapcaptt |
| ABO | 2.75E-147 | creatinine albumin alp fviii tissueplasactiv vonwillebrandfactor |
| ABO | 2.86E-147 | creatinine albumin alp fviii tissueplasactiv vonwillebrandfactor |
| ABO | 3.47E-147 | creatinine alp fviii tissueplasactiv vonwillebrandfactor |
| APOA5 ABO | 2.68E-142 | alp triglycerides fviii tissueplasactiv vonwillebrandfactor |
| F12 ATP2B2 UGT1A6 | 4.48E-137 | bilirubintotal aptt |
| F7 EDEM2 | 1.56E-123 | cholesterol fvii fvix diastolic |
| F7 RORA | 1.47E-115 | cholesterol fvii fvix diastolic |
| APOA5 SERP1 CD46 MYLK UGT1A1 | 3.23E-115 | bilirubintotal triglycerides |
| F7 | 4.64E-115 | cholesterol hdlcholesterol fvii fvix diastolic |
| FGF12 ATP2B2 UGT1A4 | 8.86E-114 | bilirubintotal |
| CNOT2 NPY1R UGT1A5 | 1.42E-113 | bilirubintotal |
| TYMP UGT1A10 | 2.13E-113 | bilirubintotal |
| BCAP31 NPY1R UGT1A4 | 2.49E-113 | bilirubintotal |
| LRRC28 UGT1A6 | 3.56E-113 | bilirubintotal |
| UGT1A5 TPH1 | 4.37E-113 | bilirubintotal |
| UGT1A9 FAM161A | 6.11E-113 | bilirubintotal |
| UGT1A10 RBBP7 SCGB1A1 NEURL | 8.75E-113 | bilirubintotal |
| UGT1A5 | 3.21E-112 | bilirubintotal |
| F10 ACSS2 ANKS1B TSC2 | 7.48E-86 | fvii fvix |
| F10 ZDHHC24 APOM TEX12 ZNF259 | 3.37E-81 | hdlcholesterol triglycerides fvii fvix |
| PARD3B CYP51A1 HFE HOXA7 APOE RPL28 SURF4 | 1.97E-69 | hb mchaemoglobin alp cholesterol ldlcholesterol vonwillebrandfactor |
| C11orf74 F5 PVRL2 TIA1 C11orf10 ABCC12 | 7.84E-68 | ldlcholesterol ratioapcaptt |
| F5 FAM71E2 KNG1 SCAF1 RFC1 SCUBE1 | 1.59E-67 | aptt ratioapcaptt |
| CETP SLC39A14 PISD DFNB31 ART3 SEMA5B PPP2R4 ACSM3 SURF4 IQCG GTF3C5 RABGAP1L | 5.55E-67 | alp hdlcholesterol vonwillebrandfactor cornellindex cornellproduct |
| F5 TMCC1 HMHA1 MTMR10 ATG16L1 C2 TEAD2 PRKRA | 2.69E-66 | ratioapcaptt |
| FAM189B F5 ANKS1A ATG16L1 RFC1 SCUBE1 NAV3 | 4.52E-66 | ratioapcaptt |
| PKP3 F5 HS6ST3 DFNB59 | 5.53E-64 | ratioapcaptt |
| F5 FAM63B ST3GAL1 CALCA GNA14 | 1.37E-62 | ratioapcaptt |
| APOA5 LDLRAD3 ESR2 GPRC6A HFE HMGA1 SURF4 | 3.70E-62 | mchaemoglobin alp triglycerides plasmaviscosity vonwillebrandfactor |
| CHKB HFE MUC1 SURF4 | 3.30E-61 | mchaemoglobin platelets lymphocytes magnesium alp hdlcholesterol vonwillebrandfactor printerval cornellindex |
| CHRNA3 F12 APOE GNA14 | 1.38E-55 | phosphate cholesterol ldlcholesterol fibrinclot aptt |
| SURF1 SURF4 | 9.36E-54 | creatinine alp fviii tissueplasactiv vonwillebrandfactor tnfa |
| FPGT-TNNI3K ERI2 LRRC71 ALCAM NCAPD3 THUMPD3 IL16 ITGA2 MYH1 TRAF1 CAMK2G | 2.91E-52 | qrsduration cornellindex cornellproduct |
| CHGA UCN3 SERP1 IAPP APOE PDE7A PLK1 ASXL2 TPMT BUD13 LIPG | 5.82E-46 | ldlcholesterol triglycerides qrsvoltagesum qrsvoltageprod |
| YKT6 CHGA APOA5 CTH ESR2 MYLK PDE7A PRR5-ARHGAP8 PTPRE SLC2A2 LEFTY2 CAMK2G DLGAP1 BAG5 | 1.25E-42 | triglycerides qrsvoltagesum qrsvoltageprod |
| CETP F12 NSF ARHGEF3 | 2.63E-42 | hdlcholesterol triglycerides aptt |
| FPGT-TNNI3K ACTR3 ABCC2 CNR1 ALCAM PPP1R14B CXCR3 ITGA2 PPARG STX17 BBS4 ACSM3 BRCA2 LTBP4 | 2.64E-41 | qrsduration cornellindex cornellproduct |
| LTB4R F12 MCC ROCK1 USP9X BUD13 TMEM132C PAN2 | 1.37E-40 | triglycerides fibrinclot aptt |
| FAF1 APOA5 ESR2 IL10RA ILF3 BAD ACSM3 TNFRSF10C IL18RAP EIF2B3 | 6.47E-40 | triglycerides qrsvoltagesum qrsvoltageprod |
| HIBADH UCN3 GPR62 DOM3Z FOXJ1 ANGPTL3 PON3 PRTFDC1 PVRL2 RBBP7 STAR TPM1 C11orf9 SLC37A3 ZNF259 GPR52 | 5.74E-39 | hb ldlcholesterol triglycerides |
| SPINK5 NISCH ERI2 ADM CTF1 DOCK2 E2F3 ALCAM POU2F3 NRG1 ITPR1 ABCB4 TYMS ZIC3 SLC39A7 IQCG | 9.76E-38 | qrsduration cornellindex cornellproduct |
| HRG MUC1 | 4.00E-33 | haematocrit magnesium correctedcalcium totprotein bc2betacarotene fvix aptt ratioapcaptt qtinterval |
| CAP2 CDYL2 CPT1B GCNT2 ANGPTL3 MLXIPL PVRL2 TAGLN CASP2 | 4.46E-30 | basinophils ldlcholesterol triglycerides tissueplasactiv |
| ABCC4 ERI2 COL11A2 CELSR2 NUP210 GATA4 GPX2 TBX21 HTR1D INSIG1 LMAN1 LCNL1 MATN2 MMP26 ABCG4 POPDC3 SLC2A2 TRAK2 TBXAS1 SUMO1 TRPM8 GAS7 | 4.78E-29 | cholesterol ratioapcaptt tnfa qrsduration lvmirautaharju lvmifhuwez qrsvoltagesum qrsvoltageprod |
| CETP ERBB3 ARHGAP8 ALOX15B GRID2 PRRT4 ART3 PDE6B TMEM19 STIM1 TUFM CALCA CALCR PER3 GTF3C5 | 1.95E-28 | hdlcholesterol cornellproduct |

Legend: This table shows the association values in CCA multiple gene / multiple phenotype analysis. The first column shows the phenotypes, the second column represents the CCA association value and the thirst column shows the genes associated.
