# Supplementary material for: Canonical Correlation Analysis for Gene-Based Pleiotropy Discovery
Source: PLoS Comput Biol. 2014 Oct 16;10(10):e1003876. doi: 10.1371/journal.pcbi.1003876 (PMC4199483; doi:10.1371/journal.pcbi.1003876)
Supplement: Table S7 — Glossary of phenotypes. Complete name of the phenotype. (DOC) [file pcbi.1003876.s007.doc]

Table S7: Glossary of phenotypes

| Name | Description |
| --- | --- |
| 2ngadiponectin | Adiponectin in ug/ml |
| 2ugadiponectin | Adiponectin in ug/ml |
| adiponectin | Adiponectin in ug/ml |
| albumin | Serum albumin concentration in g/l |
| alp | Alkaline Phosphatase in U/l |
| alt | Alanine aminotransferase in U/l |
| aptt | Activated partial thromboplastin time (APTT) in seconds |
| ast | Aspartate transaminase in U/l |
| basinophils | Basinophils (x10E3/ml) |
| bc1betacarotene | First Beta-carotene assay measurement |
| bc2betacarotene | Second Beta-carotene assay measurement |
| bilirubintotal | Bilirubin |
| bminew | BMI. Body Mass Index |
| c1vitamin | First vitamin C assay measurement in uM |
| c2vitamin | Second vitamin C assay measurement in uM |
| calcium | Serum calcium concentration in mmol/l |
| cholesterol | Serum cholesterol concentration in nmol/l |
| cornellindex | Cornell Voltage Index |
| cornellproduct | Cornell Product. Product of Cornell Voltage and QRS duration |
| correctedcalcium | Corrected serum calcium |
| creactiveprotein | C- reactive protein |
| creatinine | Serum creatinine concentration in umol/l |
| cytil18 | Interleukin 18 in pg/ml |
| cytmmp9 | Matrix metallopeptidase 9 |
| cytscd40l | CD 40 protein |
| cyttnfa | TNF A (Tumor Necrosis Factor Alpha) in pg/ml |
| CVD | Factor variable indicating if patient has cardiovascular diseases |
| ddimer | D-dimer in ng/ml |
| diastolic | Diastolic blood pressure |
| e1vitamin | First vitamin E assay measurement in uM |
| e2vitamin | Second Vitamin E assay measurement in uM |
| eosinophils | Eosinophils(x10E3/ml) |
| fibrinclot | Fibrinogen clotting assay in g/L |
| fvii | FVII in iu/dl |
| fviii | FVIII in iu/dl |
| fvix | FIX in iu/dl |
| ggt | Gamma-glutamyl transpeptidase level in U/l |
| glucose | Fasting glucose concentration |
| haematocrit | Haematocrit reading from 0.00 to 0.01 |
| hb | Haemoglobin level (g/dl) |
| hba1c | Mean celular haemoglobin concentration (g/dl) |
| hdlcholesterol | Serum HDL cholesterol concentration in nmol/l |
| homascore | HOMA insulin resistant: HOMA score derived from product of fasting glucose |
| il18 | Interleukin 18 in pg/ml |
| il6 | Interleukin 6 in pg/ml |
| ldlcholesterol | Serum LDL cholesterol concentration in nmol/l |
| leptin | Leptin in ng/ml |
| lvmifhuwez | Huwez ECG derived index |
| lvmirautaharju | Rautaharju ECG derived index |
| lymphocytes | Lymphocytes (x10E03/ml) |
| magnesium | Serum magnesium concentration in mmol/l |
| mchaemoglobin | Haemoglobin level (g/dl) |
| mcvolume | Mean cell volumen |
| monocytes | Monocytes (x10E03/ml) |
| mpvolume | Mean platelet volume |
| neutrophils | Neutrophils (x10E03/ml) |
| nwinsulin | Insulin mu/l |
| phosphate | Serum phospate concentration in mmol/l |
| plasmaviscosity | Plasma viscosity |
| platelets | Platelets (x10E03/ml) |
| potassium | Serum potassium concentration in mmol/l |
| printerval | PR interval in ECG |
| qrsduration | QRS duration in ECG |
| qrsvoltageprod | QRS voltage product in ECG |
| qrsvoltagesum | QRS voltage sum in ECG |
| qtcinterval | QTC interval in ECG |
| qtinterval | QT interval in ECG |
| ratioapcaptt | Activated procein C and Activated partial thromboplastin time (APTT) ratio |
| rbcount | Red blood cell (erythrocyte) count (x10E06/ml) |
| sodium | Serum sodium concentration in mmol/l |
| sokolowlyn | Sokolowlyl index derived from ECG |
| standingheight | Standing height in mm |
| systolic | Systolic blood pressure |
| tissueplasactiv | Tissue plasminogen activator |
| tnfa | TNF A (Tumor Necrosis Factor Alpha) in pg/ml |
| totprotein | Total serum protein concentration in mmol/l |
| triglycerides | Tryglicerides |
| urate | Serum urate concentration in mmol/l |
| urea | Serum urea concentration in mmol/l |
| vonwillebrandfactor | Von Willebrand Factor |
| wbcount | White blood cell count (x10E03/ml) |
| whnewratio | Waist hip ratio |
